# Elucidating *Escherichia Coli* O157:H7 Colonization and Internalization in Cucumbers Using an Inverted Fluorescence Microscope and Hyperspectral Microscopy

**DOI:** 10.3390/microorganisms7110499

**Published:** 2019-10-28

**Authors:** Yeting Sun, Dan Wang, Yue Ma, Hongyang Guan, Hao Liang, Xiaoyan Zhao

**Affiliations:** 1 College of Food Science, Shenyang Agricultural University, Shenyang 110866, China; syt_nercv@163.com (Y.S.); guanhongyang1@163.com (H.G.); 2 Beijing Vegetable Research Center, Beijing Academy of Agriculture and Forestry Science, Beijing Key Laboratory of Agricultural Products of Fruits and Vegetables Preservation and Processing, Key Laboratory of Vegetable Postharvest Processing, Ministry of Agriculture and rural affairs, Beijing 100097, China; wangdan@nercv.org (D.W.); mayue@nercv.org (Y.M.); 3 Longda Food Group Company Limited, Shandong, Jinan 265231, China; fpl2015@163.com

**Keywords:** cucumbers, *Escherichia coli* O157:H7, hyperspectral microscopy, colonization, internalization

## Abstract

Contamination of fresh cucumbers (*Cucumis sativus* L.) with *Escherichia coli* O157:H7 can impact the health of consumers. Despite this, the pertinent mechanisms underlying *E. coli* O157:H7 colonization and internalization remain poorly documented. Herein we aimed to elucidate these mechanisms in cucumbers using an inverted fluorescence microscope and hyperspectral microscopy. We observed that *E. coli* O157:H7 primarily colonized around the stomata on cucumber epidermis without invading the internal tissues of intact cucumbers. Once the bacterial cells had infiltrated into the internal tissues, they colonized the cucumber placenta and vascular bundles (xylem vessels, in particular), and also migrated along the xylem vessels. Moreover, the movement rate of *E. coli* O157:H7 from the stalk to the flower bud was faster than that from the flower bud to the stalk. We then used hyperspectral microscope imaging to categorize the infiltrated and uninfiltrated areas with high accuracy using the spectral angle mapper (SAM) classification method, which confirmed the results obtained upon using the inverted fluorescence microscope. We believe that our results are pivotal for developing science-based food safety practices, interventions for controlling *E. coli* O157:H7 internalization, and new methods for detecting *E. coli* O157:H7-plant interactions.

## 1. Introduction

Fresh cucumbers (*Cucumis sativus* L.) are widely consumed across the globe as they are refreshing and healthy [1]. However, they are common vehicles of foodborne illnesses; field contamination or cross-contamination during processing with pathogenic microorganisms can occur, leading to a potential safety problem as fresh cucumbers are mostly eaten raw [2]. *E. coli* O157:H7 is one of the most common foodborne pathogens that reportedly contaminates fresh produce via contaminated irrigation water and soil/manure particles [3,4]; the strain can also contaminate them during their processing, distribution, marketing, and/or handling during both pre- and post-harvest stages [5]. A disease event of enterohemorrhagic *E. coli* infection in Germany was reportedly attributed to fresh cucumbers grown in Spain [2]. It is noteworthy that *E. coli* O157:H7 can survive and/or grow in a range of minimally processed cucumbers, which upon ingestion can adversely affect human health and also cause economic losses [6,7,8].

Studies have reported that *E. coli* O157:H7 can attach to the surface, stem scar tissue, and crevices of fresh vegetables, and vegetables with a rough surface are evidently more prone to *E. coli* O157:H7 colonization [9,10]. Moreover, *E. coli* O157:H7 cells apparently display a strong stomatal tropism to the guard cells of arugula, spinach, and lettuce [11,12]. The bacterium can further infiltrate into the internal tissues via wounds and natural openings [13,14]. Currently, most such studies have been conducted on leafy vegetables [15]. The mechanisms underlying the colonization of cucumbers by *E. coli* O157:H7 remain poorly documented, and there is no visual evidence either; moreover, no studies have yet been conducted to explore how *E. coli* infects cucumbers upon internalization. Hyperspectral microscope imaging (HMI) has been successfully used for the rapid identification and classification of Gram-positive and -negative foodborne pathogenic bacteria [16]. In addition, a nondestructive, noncontact hyperspectral imaging technique has been reported for the detection and identification of six non-O157 Shiga-toxin-producing *E. coli* bacteria cultivated on solid agar [17].

In this study, we investigated the mechanisms underlying *E. coli* O157:H7 colonization and internalization in fresh cucumbers using an inverted fluorescence microscope and hyperspectral microscopy. Our results provide visual evidence pertaining to the critical role of vascular bundles in pathogen internalization. We believe that our findings should provide useful information for science-based food safety. 

## 2. Results and Discussion

### 2.1. Colonization Mechanism in Intact Cucumbers

**Figure 1:**
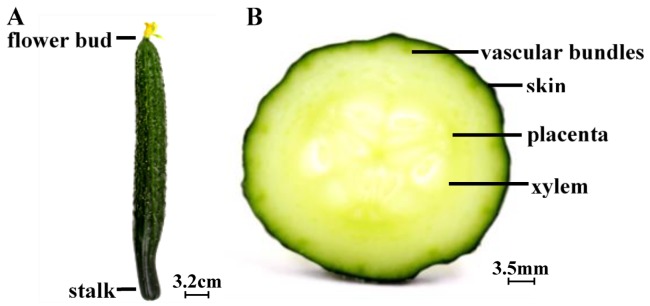
Optical images of cucumber tissue: (**A**) intact and (**B**) cross-sectional slice with three carpels.

**Figure 2:**
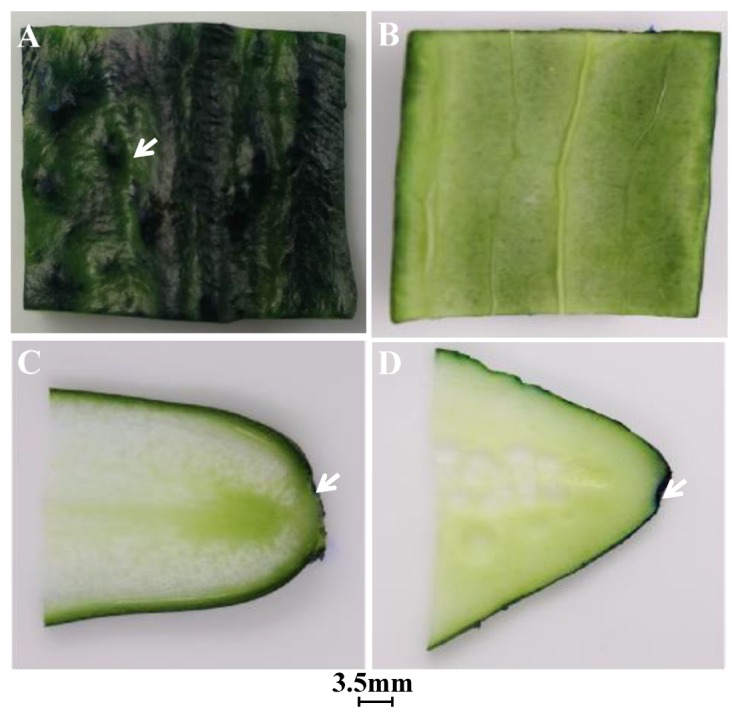
Dye uptake by intact cucumbers: (**A**) longitudinal optical image of the dye on cucumber epidermis; (**B**) longitudinal optical image of the dye on the back of the epidermis; (**C**) longitudinal optical image of dye uptake into the stalk; and (**D**) longitudinal optical image of dye uptake into the flower bud. White arrows point toward the dye. The scales of Figure A, B, C and D are the same.

We used the cucumber epidermis, stalk, and flower bud for our analyses (Figure 1). As evident from Figure 2A, the fruit skin in cucumber had more multicellular, nonglandular trichomes [18], the dye settled in the wrinkles and multicellular, nonglandular trichomes on the epidermis indicating that it barely infiltrated into the internal tissues of cucumbers (Figure 2B). Furthermore, Figure 2C,D, respectively, show the dye infiltrated into the cucumber stalk and flower bud [19], illustrating that the dye scarcely infiltrated into the internal tissues via the cucumber stalk and flower bud. This observation could also be attributed to the fact that the small amount of MB dye that infiltrated into the internal tissues was invisible to the naked eye. Thus, we used an inverted fluorescence microscope to accurately evaluate *E. coli* O157:H7 internalization.

**Figure 3:**
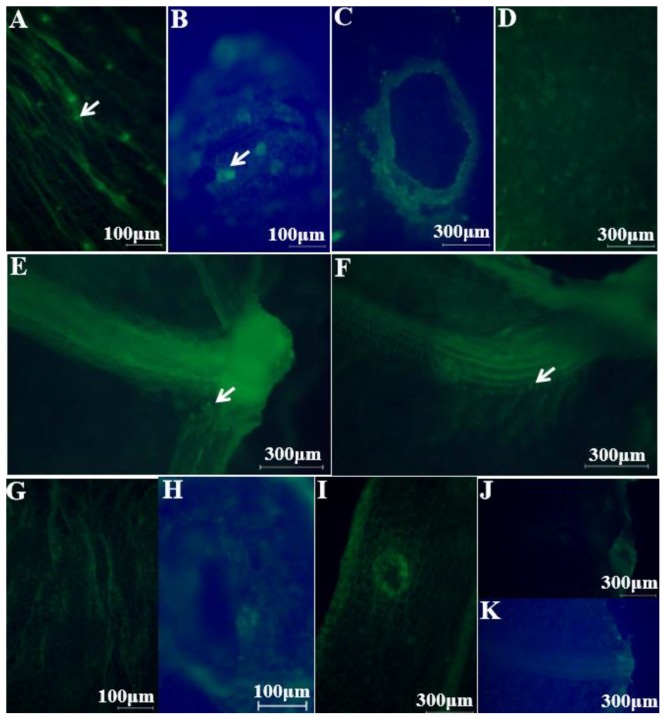
Colonization of intact cucumbers by green fluorescently labeled *Escherichia coli* O157:H7 cells: (**A**) green fluorescently labeled *E. coli* O157:H7 cells colonizing the wrinkles on the intact cucumber epidermis (100×) and (**B**) around the stomata (100×); (**C**) fluorescent image of the polycytoma of the epidermis (40×) and (**D**) of the back tissue of the epidermis (40×); (**E**) green fluorescently labeled *E. coli* O157:H7 cells that infiltrated via the stalk (40×) and (**F**) the flower bud (40×); (**G**‒**K**) are the controls of uninoculated cucumber tissues of (**A**‒**C**, **E**‒**F**), respectively. White arrows point toward the *E. coli* O157:H7 cells.

**Figure 4:**
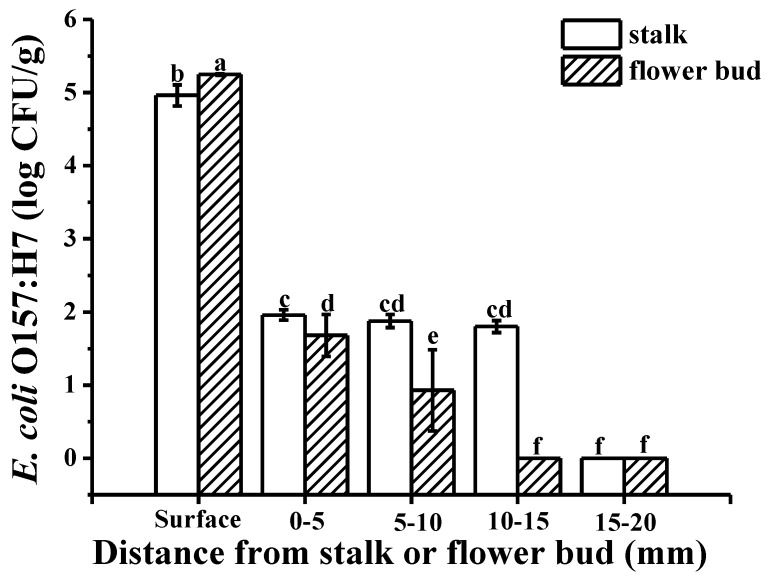
The plate count results showing *E. coli* O157:H7 colonization on the surface and upon infiltration via the stalk and flower bud. Different letters indicate statistically significant differences (*p* < 0.05).

The green fluorescently labeled cells of *E. coli* O157:H7 colonized the wrinkles on the intact cucumber epidermis (Figure 3A) and around the stomata (Figure 3B), without infiltrating into the multicellular, nonglandular trichomes (Figure 3C) and internal tissues (Figure 3D). During the picking process, wounds can form around the cucumber stalk and flower bud, resulting in the exposure of the internal tissues, thereby facilitating pathogen penetration [20]. Figure 3E,F show that some green fluorescently labeled cells of *E. coli* O157:H7 infiltrated into the internal tissues via the wounds around the stalk and flower bud, respectively; more cells seem to have infiltrated via the stalk than via the flower bud. Figure 3G–K are the controls for uninoculated cucumber tissues. The plate counts provided additional evidence regarding bacterial cell internalization *E. coli* O157:H7 showed a significant difference in colonization at certain distances (*p* < 0.05). A higher number of *E. coli* O157:H7 cells colonized the epidermis, less invading the internal tissues of the cucumber through the stalk and flower bud (Figure 4). This might be due to the abundant stomata and wrinkles present on the epidermis. Previous studies have indicated that bacteria aggregate near open stomata [21]; enterohemorrhagic *E. coli* O157 cells were reported to attach to leaf stomata using the fT3SS apparatus. The plate counts displayed that *E. coli* O157:H7 colonization was more prominent on the epidermis of the cucumber flower bud than the stalk, which could be due to the presence of more wrinkles on the flower bud surface. Studies have reported that surface roughness plays a major role in bacterial colonization; for example, a study showed that *E. coli* O157:H7 densely colonized the wrinkled surface of spinach leaves [10]. Figure 4 shows that, in comparison to the flower bud, more *E. coli* O157:H7 cells infiltrated via the stalk; the infiltration decreased with an increase in the distance from the stalk to the flower bud. The highest (average 1.96 log CFU/g) infiltration was observed at 0–5 mm below the stalk, decreasing to 0 log CFU/g at 15–20 mm below the stalk. In contrast, the highest (average 1.68 log CFU/g) infiltration was observed at 0–5 mm below the flower bud, decreasing to 0 log CFU/g at 10–15 mm below the flower bud. This result indicated that the cucumber stalk is a significant entry point for *E. coli* O157:H7. A wound in the stalk can provide access to bacterial cells to the internal tissues of cucumbers, serving as a gateway for bacterial internalization [9].

### 2.2. Colonization Mechanism in Crosscutting Cucumbers

**Figure 5:**
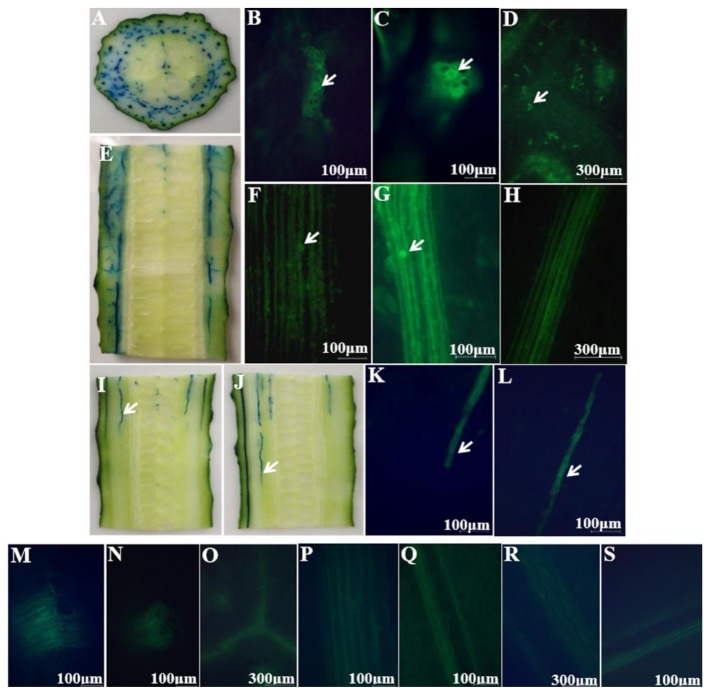
Dye uptake and colonization of sliced cucumbers by green fluorescently labeled cells of *E. coli* O157:H7: (**A**) transverse optical image of dye uptake into the tangent plane of cucumbers; (**B**) transverse sectional images illustrating the infiltration of green fluorescently labeled *E. coli* O157:H7 cells into the vascular bundles (100×), (**C**) xylem vessels (100×), and (**D**) placenta (40×); (**E**) longitudinal optical image of dye uptake into the tangent plane of cucumbers; (**F**) longitudinal sectional images illustrating the infiltration of green fluorescently labeled *E. coli* O157:H7 cells into the vascular bundles (100×), (**G**) xylem vessels (100×), and (**H**) placenta (40×); (**I**) longitudinal optical images showing the dye penetration distance from the tangent plane of the cucumber stalk and (**J**) the flower bud into the internal tissues; (**K**) longitudinal sectional images depicting that green fluorescently labeled *E. coli* O157:H7 cells infiltrated into the internal tissues from the tangent plane of the stalk (100×) and (L) the flower bud (100×). White arrows point toward green fluorescently labeled *E. coli* O157:H7 cells; (**M**‒**S**) are the controls of inoculated cucumber tissues of (**B**‒**D**, **F**‒**H**, **K**‒**L**), respectively. (**K**) and (**L**) belong to the same tissue part. White arrows point toward the dye or *E. coli* O157:H7 cells.

**Figure 6:**
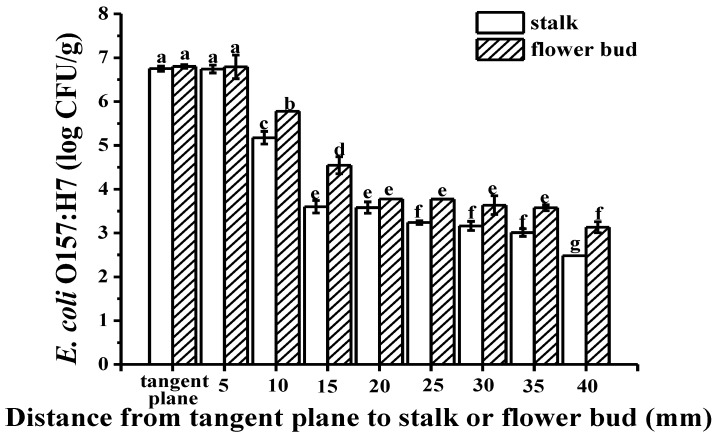
The plate count results showing *E. coli* O157:H7 colonization on the tangent plane and upon infiltration via the stalk and flower bud. Different letters indicate statistically significant differences (*p* < 0.05).

Figure 5A,E depict the transverse and longitudinal optical images of dye adsorption into the tangent plane of cucumbers, respectively; the images illustrate dye uptake into the vascular bundles, xylem vessels and placenta. Similarly, inverted fluorescence microscope images of the tangent plane (Figure 5B–D,F–H; images captured at different magnifications) further illustrated that the green fluorescently labeled cells of *E. coli* O157:H7 had infiltrated into the vascular bundles, xylem vessels, and placenta. This observation could be attributed to the motility of *E. coli* O157:H7, which occurs via peritrichous flagella, thereby promoting bacterial colonization [22]. As evident from Figure 5I,J, the dye penetration distance from the tangent plane of the stalk into the internal tissue was longer than from that of the flower bud. Fluorescent images (Figure 5K,L) also confirmed that *E. coli* O157:H7 cells had infiltrated into the xylem vessels from the tangent plane of the stalk farther than from that of the flower bud. The mechanism underlying the internalization of bacterial cells into fresh cucumbers is similar to that reported for tomatoes; *E. coli* O157:H7 cells reportedly migrate along the xylem vessels of tomatoes, resulting in widespread contamination [9]. Figure 5M‒S are the controls for uninoculated cucumber tissues. The plate counts further confirmed the differences in *E. coli* O157:H7 colonization (Figure 6); there was no difference in bacterial colonization between the tangent plane of the stalk and flower bud (*p* < 0.05). *E. coli* O157:H7 invaded the internal tissues more prominently from the flower bud than from the stalk, with the infiltration gradually decreasing with an increase in the distance from the stalk and flower bud. There exists a connected pore network in the cucumber tangent plane that facilitates bacterial penetration. Bacterial transport is caused primarily by flow due to the pressure gradient, and the movement or diffusion of water. Moreover, in general, nutrients and moisture are transported upward from the root in xylem vessels [23], i.e., from the tangent plane to the flower bud, which may make bacterial deeper and more extensive infiltration.

### 2.3. Visualizing Colonization Mechanisms Using HMI

**Figure 7:**
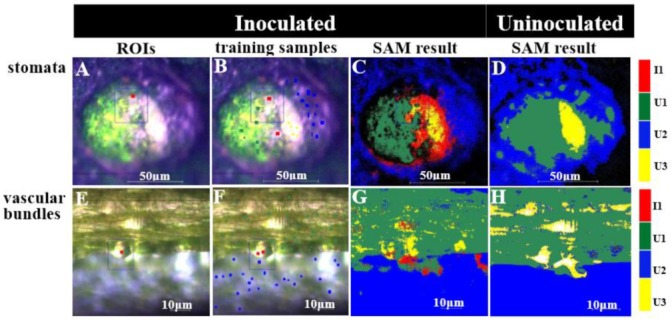
Hyperspectral microscopy images of inoculated and uninoculated cucumbers in visible and near infrared hyperspectral bands: (**A**) ROIs of cucumber stomata inoculated with *E. coli* O157:H7, 40×; (**B**) training samples of the stomata inoculated with *E. coli* O157:H7 on the image, 40×; (**C**) classification map of infiltrated, uninfiltrated, and entire areas of the stomata inoculated with *E. coli* O157:H7, 40×; (**D**) classification map of cucumber stomata uninoculated E. coli O157:H7, 40×; (**E**) ROIs of cucumber vascular bundles inoculated with *E. coli* O157:H7, 100×; (**F**) training samples of the vascular bundles inoculated with *E. coli* O157:H7 on the image, 100×; (**G**) classification map of infiltrated, uninfiltrated, and entire areas of the vascular bundles inoculated with *E. coli* O157:H7, 100×; and (**H**) classification map of cucumber vascular bundles uninoculated *E. coli* O157:H7, 100×.

Figure 7 provides further visual evidence pertaining to the colonization of cucumber tissues by *E. coli* O157:H7. Figure 7A–C show the hyperspectral microscopy images of the stomata and Figure 7E–G show those of the vascular bundles (520 × 696 pixels). Figure 7A,E show the tissues infected and uninfected by *E. coli* O157:H7; three ROIs were captured: red corresponds to infected tissue areas, green corresponds to uninfected tissue areas, and blue represents the entire selection. The spatial distribution of the test samples is shown in Figure 7B,F. Details pertaining to the training samples have been presented in Table 1. The classification map constructed using the SAM method is shown in Figure 7C,G. Figure 7D,H are the classification maps of uninoculated cucumber tissues. Intraclass spectral variability for the test samples has been presented in Table 2. The I1, U2, and U3 classes showed relatively large mean intra-class spectral angles with a small standard deviation, implying that they had higher spectral variations. The stomatal test samples showed relatively large mean intra-class spectral angles in comparison to the vascular bundle test samples. The inter-class spectral angles between each cucumber tissue area showed relatively large inter-class spectral angles, except that the spectral angles of the I1 and U3 classes were relatively small. Furthermore, the inter-class angles of I1, U1, and U2 classes were larger than the corresponding intra-class angles. This implied that most classes were highly distinguishable. The SAM classification method could thus be effectively used to distinguish between infected and uninfected tissue areas. Separability analyses was further performed using the Jeffries-Matusita (J-M) distance [24,25]. The J-M distance between a pair of probability distributions (spectral classes) converges asymptotically to 2.0 as a function of the distance between classes means. When the J-M distance was >1.85, the separability between the two classes was considered good, and when it was <1.0, the two classes were considered to be very poorly separated. Herein the J-M distances for all classes were very well separable (i.e., >1.85; Table 2). With regard to the classification accuracies from different cucumber tissue areas, the producer and user accuracies, overall accuracy, and Kappa coefficient for the I1 class produced by the SAM method were higher (Table 2). The overall accuracy of the vascular bundle test samples was 15.06% higher than that of the stomata test samples. The results produced by the SAM method were statistically accurate, and all results validated the effectiveness of the SAM classification method. Figure 7C,G depict the classification results using the SAM method, showing differences between the vascular bundle target classes and background. The classification map distinguished the infected areas compared to the uninoculated cucumber tissue samples. Less pixels of sterile areas have misidentified as the I1 class, and this finding was consistent with the results presented in Table 2. The U1, U2, and U3 classes were more accurately identified in the stomata and vascular bundles, and there was less omission for the I1 class. The color matching of sample classification was acceptable. The region of *E. coli* O157:H7 colonization could be observed as changes in coloration.

**Figure 8:**
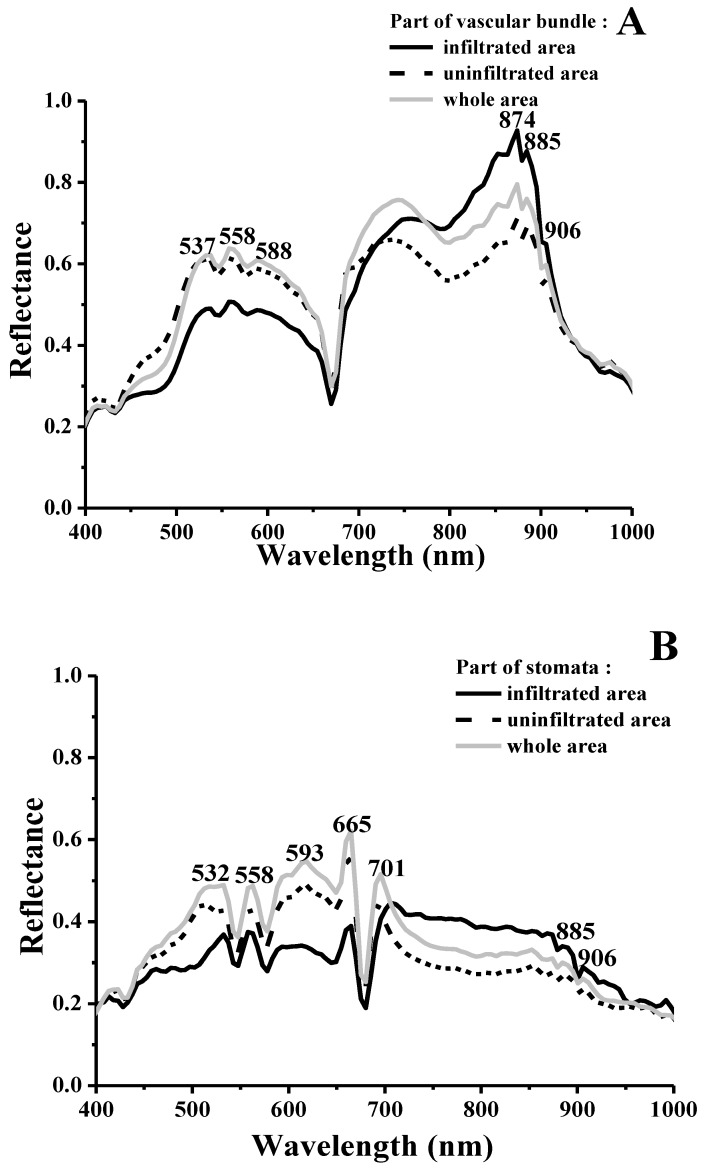
Mean spectral reflectivity characteristics within the selected region of interests for the vascular bundle (**A**) and stomata (**B**) representing infiltrated, uninfiltrated, and entire areas.

Figure 8 presents the average reflectance spectra of *E. coli* O157:H7 in the ROIs of cucumber stomata and vascular bundles, covering the effective spectral range of 400–1000 nm. Good separation between infected and uninfected areas was observed in the visible and near-infrared range; all areas exhibited high intraclass variability in both spectral magnitudes. However, the spectral shape and distribution of each infected tissue was more similar in the range of 400–670 nm; the average reflectance of infected areas around 532 nm, 558 nm, and 593 nm was higher than that of other areas. There were considerable differences in spectral characteristics of different cucumber tissues infiltrated by *E. coli* O157:H7 in the range of 670–1000 nm. The reflectance after stomata infiltration gradually decreased from 701 nm in this spectral range; however, the reflectance of the vascular bundle slowly increased from 789 nm. This might be due to the different spectral absorption of different tissues.

## 3. Materials and Methods 

### 3.1. Cucumbers

Fresh green cucumbers with three carpels, purchased from a local supermarket, were used. They were sorted to discard those with any signs of decay and visible defects. The cucumbers used for further experiments were stored at 10 °C for one day [26]. Figure 1 depicts the cucumber species used in this study, being similar to the one studied by Dan et al. [27].

### 3.2. Inoculum Preparation

A nonpathogenic strain of *E. coli* O157:H7 (ATCC 700728) was obtained from Beina Chuanglian Biotechnology Institute (Beijing, China). *E. coli* O157:H7 was activated by two successive transfers in tryptic soy broth (Aoboxing Bio-tech Co., Ltd., Beijing, China) at 37 °C for 24 h. Activated cells were grown overnight in modified *E. coli* (EC) broth (Land Bridge Technology Co., Ltd., Beijing, China) supplemented with 20 μg/mL novobiocin at 37 °C. Subsequently, cultures grown overnight were centrifuged at 9000 rpm for 10 min, and the sedimented pellet was washed once with sterile deionized water (SDW) and then diluted 1:10 in sterile Butterfield’s buffer to obtain a final *E. coli* O157:H7 concentration of approximately log 6–7 CFU/mL (optical density at 600 nm [OD_600_] of 0.6). 

### 3.3. Dye Infiltration into Cucumbers

Intact or transversely cut cucumbers were held in 1% methylene blue (MB) dye solution for 30 min; subsequently, we longitudinally or horizontally sliced the cucumber epidermis, stalk, flower bud, and fruit using a sterile knife. Images of the sliced surfaces were captured using a digital camera (EOS 600D, Canon Inc., Tokyo, Japan).

### 3.4. Microbiological Analyses

#### 3.4.1. Intact Cucumbers

The method used for evaluating *E. coli* O157:H7 colonization was based on the one described by Zhou et al. [9], with some modifications. Four batches of five cucumbers each were submerged in the bacterial inoculum at a depth of 34 cm for 30 min. The cucumbers were then removed from the inoculum, and the loosely attached cells were washed with SDW. A 3-cm long cucumber stalk and flower bud were transversely sliced using a sterile knife; a cylindrical plug of the core tissue beneath the stalk or flower bud was obtained by pushing a sterile stainless-steel perforator from the less contaminated cross section toward the more contaminated region of the stalk or flower bud. The core tissue plug inside the sterile stainless-steel perforator was then removed by pushing it in the reverse direction. The core tissue was sequentially sliced into 5-mm, 10-mm, 15-mm, and 20-mm segments from the stalk or flower bud to the internal edge to finally obtain four segments. A different identical blade was used for each slice to minimize the risk of contamination and experimental error. One segment of the same portion of each tissue was placed in a sterile Erlenmeyer flask (five segments in total) and mixed along with 30 mL PBS for 2 min in a stomacher blender. Then, 0.05-mL suspensions were surface plated in duplicate on sorbitol McConkey agar [28,29], followed by incubation at 37 °C for 24 h; the colonies were thereafter enumerated. We placed 10 g of the inoculated epidermis sample in a sterile Erlenmeyer flask and mixed it along with 90 mL PBS for 2 min in a stomacher blender. The filtrate was serially diluted 10-fold with PBS, and 0.05 mL diluted droplets were then plated on sorbitol McConkey agar, followed by incubation at 37 °C for 24 h. The obtained colonies were subsequently enumerated by the flat colony counting method. 

#### 3.4.2. Crosscutting Cucumbers

In a similar manner to the above, crosscutting cucumber once each were submerged in the bacterial inoculum at a depth of 0.5 cm, removed after 30 min, and washed with SDW. The cucumber epidermis was peeled with a sterile knife and the core tissue was sequentially cut into 1-mm, 5-mm, 10-mm, 15-mm, 20-mm, 25-mm, 30-mm, 35-mm, and 40-mm segments from the tangent plane to the internal tissue to finally obtain nine segments. One segment of the same portion of each tissue was placed in a sterile Erlenmeyer flask (five segments in total) and mixed along with 30 mL PBS for 2 min in a stomacher blender. The filtrate was serially diluted 10-fold with PBS, and 0.05 mL diluted droplets were plated on sorbitol McConkey agar, followed by incubation at 37 °C for 24 h. The obtained colonies were then enumerated by the flat colony counting method. 

### 3.5. Visualizing *E. coli* O157:H7 Colonization Using an Inverted Fluorescence Microscope

Cucumbers were inoculated as previously described, and the cucumber surface or internal tissue was transversely or longitudinally sliced (thickness = 0.5–1 mm) using a sterile knife. The samples were then stained with 0.05% fluorescein isothiocyanate in PBS (10 mM KH_2_PO_4_, 150 mM NaCl, pH 7.2) for 30 min [30]. Excess stain was removed by rinsing the sample with SDW, and the sample was then placed on a microscope slide and visualized under an inverted fluorescence microscope (BDS200-FL, Nanbei Instrument Equipment Co., Ltd., Zhengzhou, China). The green fluorescence of fluorescein isothiocyanate was detected using the blue excitation (B1) filter block. The primary filtering element in the B1 filter block is the set of three filters housed in the fluorescence filter cube: the excitation filter, dichroic beam splitter, and cut-off filter. Data are based on at least 10 fields of view per sample. 

### 3.6. Visualizing *E. coli* O157:H7 Colonization Using HMI

The HMI system consists of an inverted fluorescence microscope, a built-in dual CCD camera with a grating-based imaging spectrometer (SOC-710, Los alamitos, CA, USA), and a L-150A single-fiber cold-light darkfield illumination source (150 W halide lamp, Nanguang Electronics Technology Co. Ltd., Suzhou, China). The grating-based HMI system can be mounted to any microscope for biological scanning. As selected in a previous study for obtaining high-quality data and minimal distortion of the sample [31]. Herein, HMI were collected at wavelengths ranging from 400 to 1000 nanometers with a spectral resolution of 1.3 nm in order to obtain real-time research-grade results. All images acquired using the CCD camera were recorded using the HyperScanner_2.0.127 data acquisition software (Beijing AZUP scientific Co., Beijing, China); dark calibration was performed on each sample to obtain the reflectance *R* from the raw data. The hyperspectral data obtained from the cucumber sample comprised contiguous wavebands for each spatial position of a target, and each pixel contained the spectrum of that specific position, which could be regarded as a fingerprint. This fingerprint was used to illustrate the characteristic spectral curve of *E. coli* O157:H7 in the sample. The recorded images were then converted to float files using the SR analysis software (SOC-710, SOC). Subsequently, the region of interests (ROIs) on the spectral image at 462 nm, 547 nm, and 639 nm were selected using the ENVI Classic software (version 4.8, Exelis Visual Information Solutions, Inc., Boulder, CO, USA), and the raw hyperspectral data were smoothed with an ENVI Gaussian filter. For all bands, the average reflectance of each treatment sample was calculated using the Savitzky‒Golay method with a second-order polynomial [32]. The reference spectra of the known samples were used as the training sample, and each pixel spectrum was compared with all reference spectra of the known class. It can be identified whether a target pixel belongs to a user-specified category [33]. Training samples were first selected from the image and the square area (approximately 100 pixels) around each remaining pixel was selected as the test area. The SAM method was used to construct a classification map to predict the distribution of *E. coli* O157:H7. A color map was then constructed on the original spatial locations of the source pixel spectra [34]. Each type has been shown via a color bar on the right of the classification map; the map depicts a direct correlation with the spatial information pertaining to *E. coli* O157:H7 present in the sample.

### 3.7. Statistical Analyses

Data were statistically analyzed with one-way analysis of variance, and statistical analysis was performed using SPSS 20.0 (SPSS Inc., Chicago, IL, USA). Significant differences between treatments were determined using Duncan’s test, and the 5% level of significance was considered in all analyses. Image-Pro Plus 6.0 was used to process images. Hyperspectral data were processed using ENVI and plotted with Origin Pro 8.0 (OriginLab Corporation, Northampton, MA, USA).

## 4. Conclusions

In the case of intact cucumbers, *E. coli* O157:H7 colonized the stomata on the cucumber epidermis, without invading the internal tissues. Upon internalization, the bacterial cells colonized the placenta and vascular bundles (particularly the xylem vessels), migrating along the xylem vessels. Moreover, the movement rate of *E. coli* O157:H7 from the stalk to the flower bud was faster than that from the flower bud to the stalk. We could classify the infiltrated and uninfiltrated areas with high classification accuracy using the SAM method, generating visual evidence regarding the mechanisms underlying *E. coli* O157:H7 colonization and internalization in fresh cucumbers.

## 5. Highlights

> *E. coli* O157:H7 primarily colonized the stomata and wrinkles on the cucumber epidermis.

> *E. coli* O157:H7 invaded the vascular bundles and migrated along the xylem vessels.

> The movement rate of *E. coli* O157:H7 from the stalk to the flower bud was faster.

> Hyperspectral microscopy gave spatial information on *E. coli* O157:H7 in cucumbers.

## Figures and Tables

**Table 1:** Cucumber tissue training samples used in the study.

Symbol of Cucumber Tissue	Description	Stomata Training Samples (Points)	Vascular Bundle Training Samples (Points)
I1	Infiltrated area	218	200
U1	Uninfiltrated area	937	3123
U2	Dark and uninfiltrated area	1008	1884
U3	Bright and uninfiltrated area	169	140
Total		2332	5347

The training samples refer to the reference spectrum of the known samples, each pixel spectrum was compared and classified with all reference spectrum of the known class.

**Table 2:** Intra- and interclass spectral angles (in radians), J-M distance, and confusion matrix of the SAM classification (in percent) from different tissue areas.

		Stomata				Vascular Bundles			
		I1	U1	U2	U3	I1	U1	U2	U3
Mean angle		0.1659	0.2670	0.1460	0.1931	0.0928	0.0757	0.0645	0.1205
Standard deviation		0.0474	0.0726	0.0631	0.0482	0.0516	0.0554	0.0308	0.0637
PA		85.78	72.25	76.88	88.17	45.5	94.04	100	90.71
UA		75.71	100	99.74	83.71	91.92	97.25	98.64	39.94
OA		76.67				94.24			
Kappa		67.78				89.37			
Inter-class	I1	-	0.1221	0.5102	0.0208	-	-	-	-
	U1	-	-	0.5568	0.0693	0.0614	-	-	-
	U2	-	-	-	0.2633	0.4779	0.5393	-	-
	U3	-	-	-	--	0.0407	0.1021	0.2796	-
J–M distance	I1	-	1.986	2.000	1.884	-	-	-	-
	U1	-	-	1.939	1.999	1.980	-	-	-
	U2	-	-	-	2.000	1.964	1.999	-	-
	U3	-	-	-	-	1.868	1.984	1.995	-

PA, producer’s accuracy; UA, user’s accuracy; OA, overall accuracy.
